# From guidelines to action: implementation opportunities and realities

**DOI:** 10.7448/IAS.17.4.19487

**Published:** 2014-11-02

**Authors:** Wafaa El-Sadr

**Affiliations:** ICAP, Columbia University, New York, NY, USA

## Abstract

The past decade has seen notable progress in confronting the HIV epidemic. By the end of 2013, almost 13 million persons living with HIV had access to antiretroviral therapy (ART) in low- and middle-income countries (LMIC). In addition, progress has also been achieved in terms of HIV prevention with 26 LMIC achieving a 50% decrease in number of new infections including 15 countries in sub-Saharan Africa. New research findings also offer new efficacious prevention interventions that offer new opportunities for further mitigation of new HIV infections. Such progress has inspired ambitious pronouncements such as achieving “The End of AIDS” or “An AIDS Free Generation.” This progress has also motivated the development of new guidelines aimed to achieving these goals. Implementation of guideline recommendations, however, requires translation of these guidelines into actionable steps and then their successful delivery within established health services. The opportunities offered by new guidelines frequently meet the realities of the frailties of the health system. Achieving current ambitious global targets must confront the specific deficits in the health system that inhibit access and acceptability of programmes as well as hinder achievement of high quality or desired coverage.

